# Xanthogranulomatous Salpingo-Oophoritis as a Rare Presentation in Pelvic Inflammatory Disease

**DOI:** 10.7759/cureus.53693

**Published:** 2024-02-06

**Authors:** Manupriya Sharma, Renu Singh, Poojan Marwaha, Deychen Myes, Nisha Malik

**Affiliations:** 1 Pathology and Laboratory Medicine, All India Institute of Medical Sciences, Bilaspur, Bilaspur, IND; 2 Obstetrics and Gynaecology, All India Institute of Medical Sciences, Bilaspur, Bilaspur, IND

**Keywords:** chronic inflammation, pelvic inflammatory disease, pelvic mass, female genital tract, xanthogranulomatous salpingo-oophoritis

## Abstract

Xanthogranulomatous salpingo-oophoritis is an infrequent and challenging inflammatory condition of the female genital tract. It involves the destruction of the fallopian tube and ovarian tissue by infiltrating inflammatory cells comprising lipid-laden macrophages, lymphocytes, plasma cells, and multinucleated giant cells. While more commonly found in other organs like the gallbladder and kidney, its occurrence in the female genital tract is rare. We present a case of xanthogranulomatous salpingo-oophoritis in a 45-year-old woman, shedding light on its diagnostic and clinical complexities. Notably, this case features a rare histopathological finding of coexisting salpingitis isthmic nodosa (SIN) with xanthogranulomatous inflammation, adding to its uniqueness.

## Introduction

Xanthogranulomatous salpingo-oophoritis is a rare form of chronic inflammation characterized by the destruction of the normal fallopian tube and ovarian tissue due to the accumulation of inflammatory cell infiltrate comprising of lipid-laden foamy macrophages, lymphocytes, plasma cells, and multinucleated giant cells [1]. While it is more commonly associated with organs like the gallbladder and kidney, its occurrence in the female genital tract is exceptionally rare [2]. This condition can mimic malignancy on imaging and may lead to unnecessary extensive surgeries [3]. We present a case of xanthogranulomatous salpingo-oophoritis in a 45-year-old woman, emphasizing the significance of proper clinical evaluation and radiological imaging in avoiding unnecessary extensive surgeries based on suspicion of malignancy. Additionally, this case is unique in that it is associated with the coexistence of salpingitis isthmic nodosa (SIN), a previously unreported finding in conjunction with xanthogranulomatous salpingo-oophoritis.

## Case presentation

A 45-year-old woman, P3L3 (indicating three pregnancies and three live births), presented with a history of continuous dull aching pain in her lower abdomen. The pain had been of moderate to severe intensity for the past two months. There was no associated history of vaginal discharge, fever, significant weight loss, or appetite loss. Additionally, there were no reported bowel and bladder complaints. The patient had no history of other medical comorbidities.

Upon physical examination, her body mass index was 22.5 kg/m^2^, and the patient was hemodynamically stable. No lymphadenopathy was observed. The chest and cardiovascular examinations were normal. During abdominal examination, a tubectomy scar was noted, along with moderate tenderness in the lower abdomen. No palpable mass was detected on per-abdominal examination. However, during the per-vaginal examination, an ill-defined mass, measuring approximately 6x4 cm^2^, was identified in the pouch of Douglas. This mass appeared adherent to the pouch of Douglas. The same mass was felt during the per-rectal examination.

Laboratory investigations revealed a hemoglobin level of 9 g/dl and a leukocyte count of 18,000/mm^3^. Additionally, there was a moderate increase in the erythrocyte sedimentation rate (ESR), which measured 50 mm in the first hour. Her chest X-ray showed no abnormalities.

A pelvic ultrasound revealed a right complex cystic mass measuring 5x4.8x4.6 cm^3^ with increased vascularity. The tumor marker cancer antigen 125 (CA-125) was mildly elevated, measuring 47 U/ml. Serum levels of lactate dehydrogenase (LDH), alpha-fetoprotein (AFP), cancer antigen 19-9 (CA 19-9), and carcinoembryonic antigen (CEA) were within normal ranges.

With a provisional diagnosis of pelvic inflammatory disease (PID) with a tubo-ovarian mass, injectable antibiotics were initiated. However, the patient did not respond to treatment, and her pain did not subside. As a result, the patient was planned for laparotomy, during which a right tubo-ovarian mass, approximately 5x5 cm^2^ in size, was discovered, buried under dense adhesions and adherent to the bowel posteriorly and the lateral pelvic wall. The uterus, left fallopian tube, and ovary were found to be normal. Subsequently, the patient underwent a hysterectomy with a right salpingo-oophorectomy. The patient experienced an uneventful postoperative recovery and was discharged on the sixth day after surgery.

**Figure 1:**
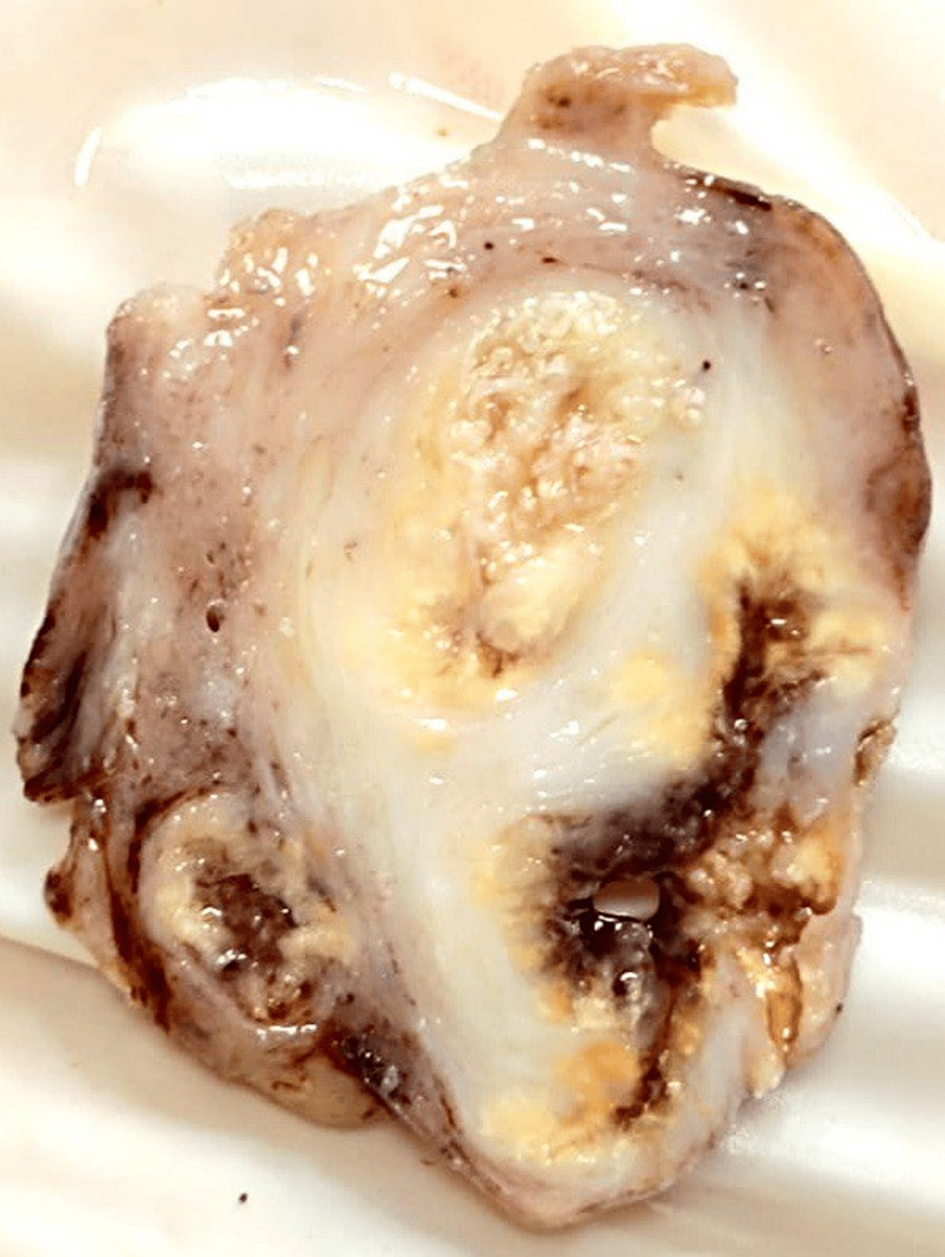
Cut section of the right adnexal mass

Macroscopically, the uterus measured 8x6x3 cm^3^, with an endometrial thickness of 0.3 cm and a myometrial thickness of 2 cm. A gross examination of the cervix revealed no remarkable abnormalities. The right adnexal mass measured 5x4x2.5 cm^3^ and displayed an irregular outer surface with focal hemorrhagic areas. Upon sectioning, the mass appeared predominantly solid, with some partly cystic areas accounting for less than 10% of the mass. Additionally, focal areas of yellowish discoloration were observed (Figure 1).

**Figure 2:**
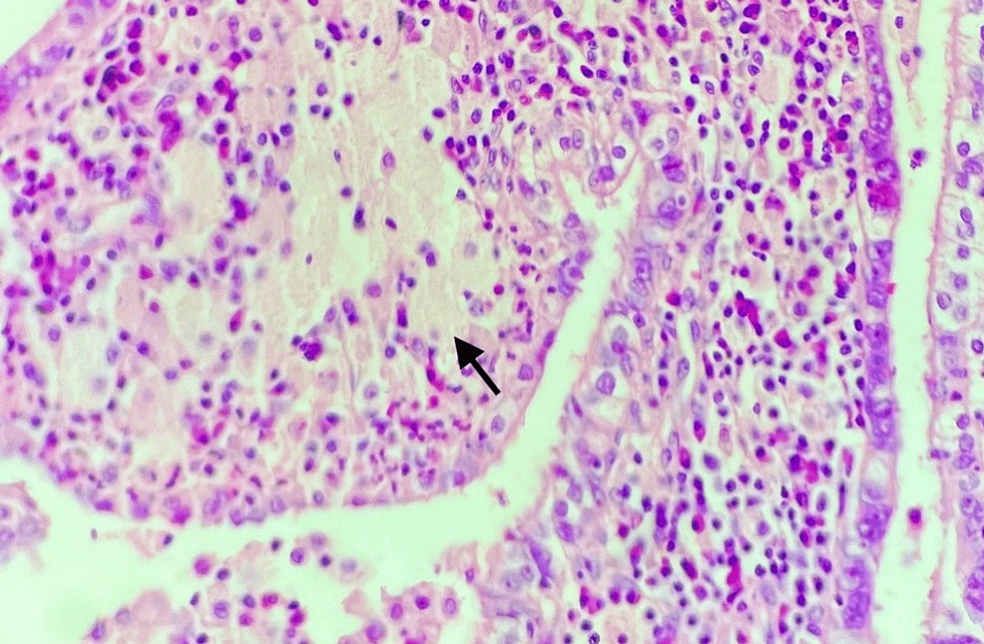
Histopathological photomicrograph of xanthogranulomatous inflammation showing distended fallopian tube plicae and replacement of fallopian tube stroma by foamy macrophages and histiocytes (H and E, 40X) Black arrow showing foamy macrophages H and E: Hematoxylin and eosin

**Figure 3:**
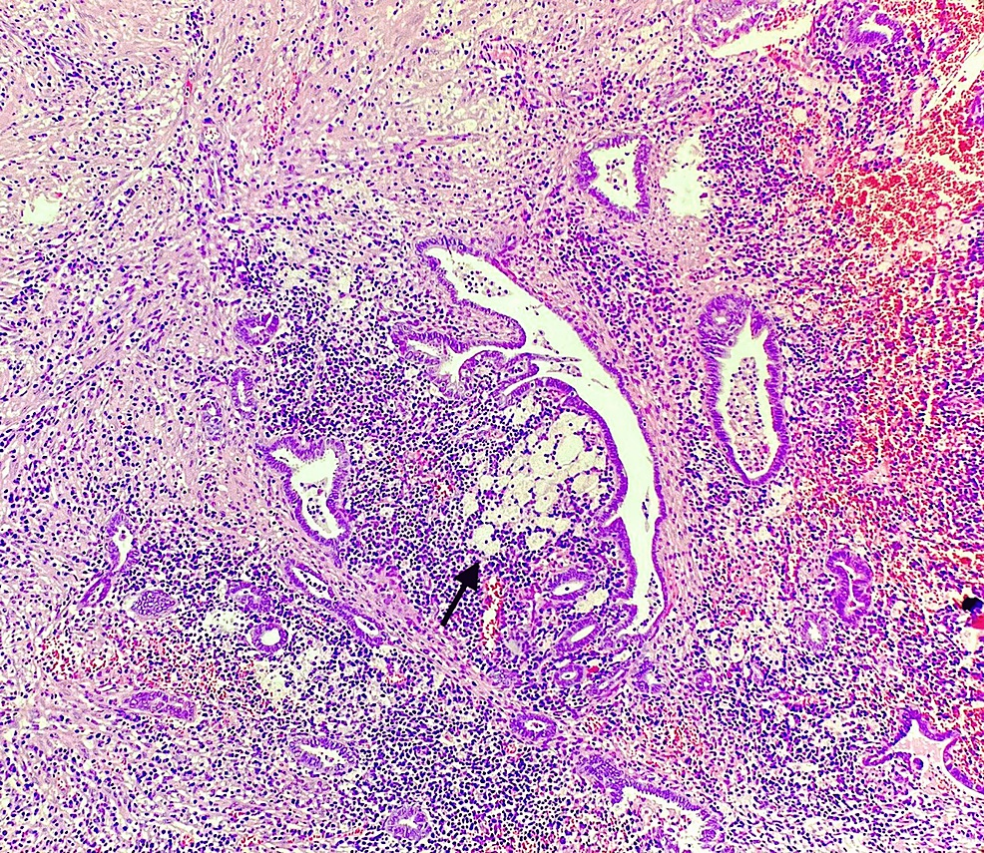
Histopathological photomicrograph showing evidence of SIN in the wall of the fallopian tube along with surrounding dense inflammation comprising of foamy macrophages and lymphoplasmacytic infiltrate (H and E, 10X) Black arrow showing foamy macrophages H and E: Hematoxylin and eosin

A microscopic examination of the sections stained with hematoxylin and eosin (H and E) was done. Sections from the tubo-ovarian mass revealed distended plicae with xanthogranulomatous inflammation (Figure 2). Special stains like acid-fast bacilli (AFB) and periodic acid-Schiff (PAS) were done and were non-contributory. Interestingly, a rare histopathological finding of coexisting SIN in the fallopian tube wall was observed (Figure 3). Dense lymphoplasmacytic infiltrate, along with macrophages, surrounded the foci of SIN.

**Figure 4:**
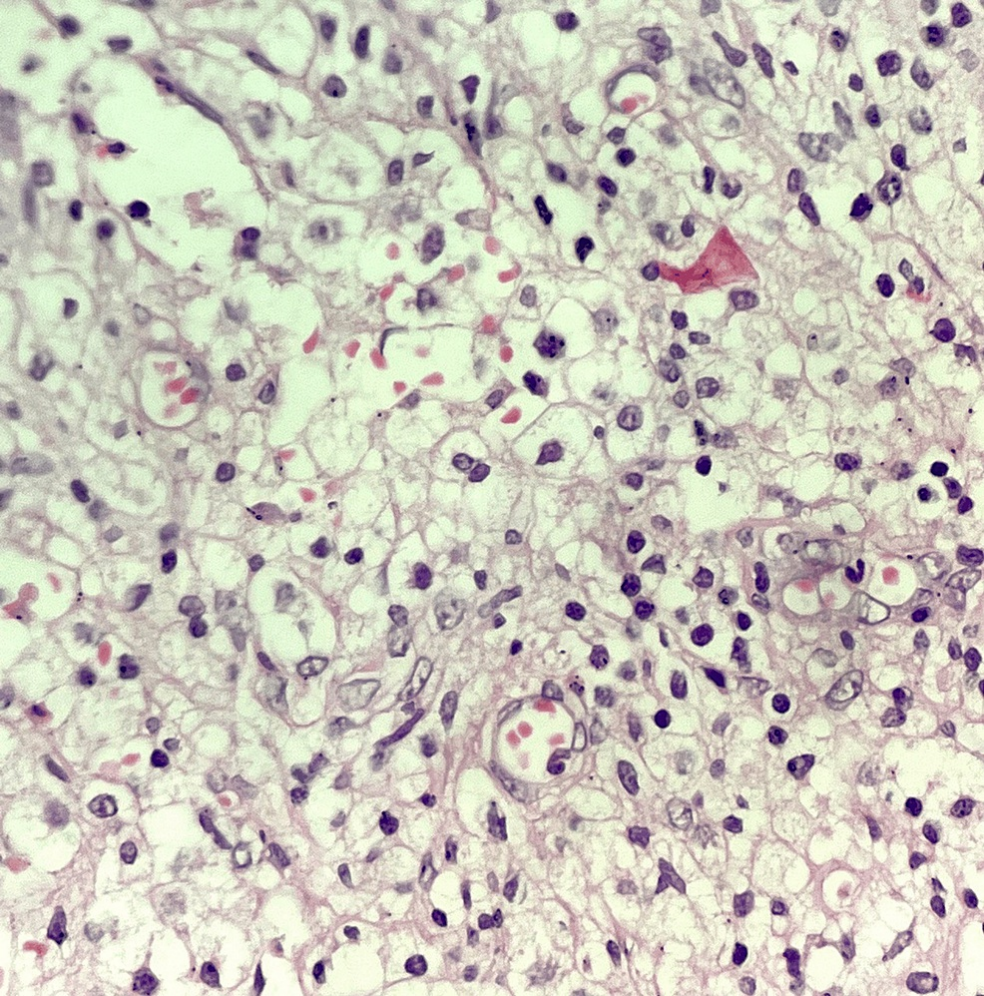
Histopathological photomicrograph of xanthogranulomatous inflammation showing replacement of ovarian stroma by foamy macrophages and histiocytes (H and E, 40X) H and E: Hematoxylin and eosin

The ovarian stroma was also replaced by sheets of macrophages, with a significant presence of plasma cells and lymphocytes (Figure 4). Notably, no granulomas or evidence of malignancy were detected. The final diagnosis of xanthogranulomatous salpingo-oophoritis was established based on these findings.

## Discussion

Xanthogranulomatous inflammation affecting the female genital tract represents a rare and distinctive form of persistent inflammation characterized by tissue damage in the affected organs. It can be associated with PID [1]. Microscopically, this condition is characterized by the presence of numerous lipid-laden foamy macrophages, along with a mixture of lymphocytes and plasma cells, which are hallmark features of xanthogranulomatous inflammation, sometimes accompanied by multinucleated giant cells [2]. While xanthogranulomatous salpingo-oophoritis is relatively infrequent in the female genital tract, it is crucial to recognize its potential association with PID [3]. Patients diagnosed with xanthogranulomatous salpingo-oophoritis typically fall within the age range of 21 to 75 years, with a mean age of 45 years [4,5]. Interestingly, the condition has even been reported in the youngest patients, with a case documented in a two-year-old child by Tanwar et al. [6].

The precise etiology of xanthogranulomatous salpingo-oophoritis remains unclear, but it appears to be associated with conditions that lead to the formation of foam cells. These conditions may include PID, endometriosis, ineffective antibiotic treatments, abnormalities in lipid metabolism, the use of intrauterine contraceptive devices, radiotherapy, and the ineffective clearance of bacteria (such as *Escherichia coli*, *Proteus* species, *Staphylococcus aureus*, *Bacteroides fragilis*, and *Salmonella typhi*) by phagocytes [7].

The clinical presentation of xanthogranulomatous salpingo-oophoritis can vary widely among patients, including symptoms such as lower abdominal pain, fever, dysmenorrhea, dyspareunia, chronic pelvic pain, abnormal uterine bleeding, reduced appetite, the presence of an abdominal mass, and even infertility. The destructive and mass-forming nature of the disease can make it exceedingly challenging to differentiate from malignancies both in terms of clinical symptoms and radiological findings [8].

The patient presented with an adnexal mass and mildly elevated CA-125 levels in our study. However, based on the clinical presentation and vigilant approach of the clinicians, along with radiological support, an inflammatory adnexal mass was suspected in the patient. It has been seen that such masses have been frequently misdiagnosed as suspicious for malignancy. However, in this case, the patient had lower abdominal and pelvic pain with adnexal tenderness on bimanual examination. Radiology findings included a heterogeneous cystic mass with internal vascularity. Her routine investigations revealed an elevated leukocyte count of 18,000/mm^3^ with predominant neutrophilia. The CA-125 was also mildly raised. Other tumor markers were within normal limits. Based on these findings, the adnexal mass was suspected to be of an inflammatory etiology.

Subsequent histopathological examination confirmed the diagnosis of xanthogranulomatous salpingo-oophoritis. An interesting finding in this case was the coexistence of SIN within the fallopian tube wall, which, to the best of our knowledge, has not been reported before in association with xanthogranulomatous salpingo-oophoritis. In this case, one of the considered differential diagnoses for SIN was tubal endometriosis; however, in endometriosis, endometrial glands are surrounded by endometrial stroma, and the glands do not possess cilia.

The pathogenesis for SIN is typically chronic inflammation, which may provide a possible explanation for its coexistence with xanthogranulomatous salpingo-oophoritis. This case highlights the complexity of such rare conditions and the importance of meticulous diagnosis and management.

## Conclusions

In summary, our case report highlights the rarity of xanthogranulomatous salpingo-oophoritis in the female genital tract and emphasizes the need for accurate clinical assessment and radiological imaging to differentiate it from malignancy. The coexistence of SIN with xanthogranulomatous salpingo-oophoritis adds complexity to our understanding of these conditions. Further research is required to elucidate the elusive etiology of xanthogranulomatous salpingo-oophoritis, potentially linked to PID.
